# Outcomes of urethral meatal preservation ventral urethroplasty for female urethral stricture: a series of cases

**DOI:** 10.61622/rbgo/2024AO20

**Published:** 2024-03-15

**Authors:** João Vitor Quadra Vieira dos Santos, Antônio Rebello Horta Görgen, Tiago Bortolini, Gabriel Veber Moisés da Silva, Emanuel Burck dos Santos, Patric Machado Tavares, Nelson Silvonei da Silva Batezini, Tiago Elias Rosito

**Affiliations:** 1 Hospital de Clínicas de Porto Alegr Porto Alegre RS Brazil Hospital de Clínicas de Porto Alegre, Porto Alegre, RS, Brazil.

**Keywords:** Urethral stricture, Urinary bladder neck obstruction, Urethroplasty, Urological surgical procedures

## Abstract

**Objective:**

To present a series of cases with our initial experience and short-term outcomes of a modified vaginal mucosal flap urethroplasty.

**Methods:**

Patients diagnosed with urethral stricture and operated by the same operative technique between January 2012 and January 2018 were followed for at least 6 months. Uroflowmetry and clinical outcomes were evaluated.

**Results:**

Nineteen patients were included with an average age of 56.4 years, mean preoperative Qmax of 5.3 ml/s, and PVR of 101.4 mL. After 6 months of the procedure, the mean Qmax improved to 14.7 mL/s (p<0.05), PVR decreased to 47.3 mL (p<0.05), and 84.2% of all patients reported improvement in clinical self-reported symptoms. There was an improvement in symptoms such as voiding effort in 84.2% of patients, weak stream (89.5%), and recurrent urinary tract infection (85.7%). The success rate (absence of symptoms and normal Qmax with no significant PVR) of the procedure was 84.2%.

**Conclusion:**

The described technique was considered effective for the treatment of female urethra stricture, with a high clinical success rate and an objective improvement of Qmax and decrease in PVR after 6 months of the procedure.

## Introduction

Female urethral stricture (FUS) is a rare cause of bladder outlet obstruction (BOO). There is a reported incidence rate of 3-8% of BOO in women, and only 4-13% of women with BOO present with FUS.^(1)^ It is currently estimated that the actual number of patients with urethral stricture is higher, but low clinical suspicion combined with a lack of well-defined diagnostic criteria make FUS difficult to identify.^(2)^

Most FUS patients have non-specific lower urinary tract symptoms, including both emptying and storage symptoms, and develop complications associated with chronic urinary obstruction, such as recurrent urinary tract infections (UTIs) or even renal failure in rare cases.^(3,4)^

The physiopathology of FUS is not yet fully understood; however, some local inflammatory processes are considered to lead to stricture. Most cases result from a traumatic or iatrogenic cause due to prolonged urethral catheter use, pelvic radiation, childbirth complications or urethral manipulations (urethral diverticula excision, urinary incontinence surgery or endoscopic procedures).^(1)^

Urodynamically, patients with urethral stricture present with a BOO pattern, urinating with low flow and high detrusor pressures, as defined as a maximum flow rate (Qmax) less than 12 mL/s during free uroflowmetry and a detrusor pressure (Pdet.max) greater than 20 cmH_2_O during voiding in the pressure-flow study.^(5)^ Diagnosis of urethral stricture is only confirmed when there is evidence of mechanical urethral obstruction, as shown by the inability to place a urethral catheter (catheter greater than 14 Fr), imaging examination, or by an endoscopic evaluation.^(6)^

Urethral dilatation is the most commonly used treatment for FUS, but it has a low success rate, often requiring additional procedures.^(7,8)^ The mean proportion of patients with improvement of symptoms after dilatation is up to 51%, with decreasing success rates as additional dilations are performed. Despite its low success rate, urethral dilation has a low rate of complications and therefore is an attractive option for most urologists.^(9)^

Surgical repair of FUS offers the most sustained and effective results, with success rates higher than 75%, but with few cases reported in the literature and numerous techniques described.^(7)^ In our study, we used a ventral approach with vaginal flap urethroplasty, as described by Schwender in 1989,^(10)^ modified by the preservation of the urethral meatus.

Herein, we present our experience with modified ventral vaginal mucosal flap urethroplasty at our institution in 19 patients who were followed up for a minimum of 6 months.

## Methods

All patients submitted for urethroplasty for mid or proximal urethral stricture were selected for review by the same operative technique between 2012 and 2018 at our hospital. We excluded only patients who were not submitted to this technique (i.e. recurrent stricture). Our hospital is the main center of reference for urethroplasty in our state (11 million people). All procedures were performed by the same group of surgeons. Our technique of choice for primary urethroplasty has been this proposed technique since 2012, with dorsal flap with oral mucosa reserved for secondary cases. The data were collected after the study was approved by our ethics committee, having all the authors signed a term of data usage, and patients signed informed consent. The diagnosis of urethral stricture was made after clinical suspicion of BOO, in combination with urodynamic findings and physical examination. Patients with urethral narrowing were defined as women who presented with a Pdet.max >20 cmH_2_Oin a pressure-flow study, Qmax <12 mL/s during free uroflowmetry with an inability to place a urethral catheter >14Fr and micturition obstruction by voiding cystourethrogram. All patients underwent a complete urodynamic evaluation before the surgical procedure and uroflowmetry with post-void residual (PVR) 6 months after that. Electroneuromyography was used to rule out functional BOO. Urethroscopy was not a routine of care for all patients but was used accordingly to surgeon indication whenever needed to diagnose or rule out differential diagnosis. Pre- and postoperative clinical and urodynamic data were evaluated by a chart review. As the protocol of care, all patients were evaluated for lower urinary tract symptoms during follow-up visits at 2, 4, 6, and 12 months and after each year. The main outcomes analyzed were the differences between pre- and postoperative Qmax and PVR values. Success was defined as absence of lower urinary tract symptoms, with a flow rate >12 ml/s and no significant PVR on the follow-up visit at 6 month.

### Operative technique

**Figure 1:**
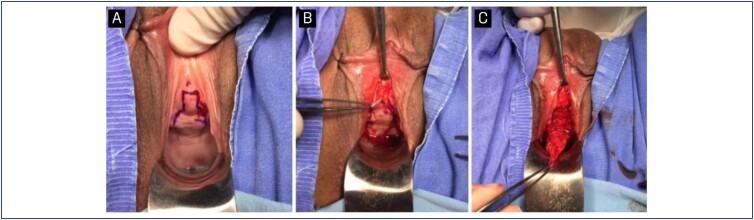
Surgery step-by-step photos

The patient was placed in a conventional lithotomy position under general anesthesia and antisepsis was achieved with aqueous chlorhexidine. Routine prophylactic antibiotics were used on anesthesia induction (cefazolin). Using a Doyen vaginal valve, the vaginal wall was exposed, and the obstruction point was identified with the use of bugie-type urethral dilatators, ranging in size from 6-12Fr. Then, an inverted U-shaped incision was made; its distal portion was 1cm from the meatus. The base of the incision had a minimum length of 2cm in order to maintain good vascularization and sustentation of the flap. The total size of the flap varied as necessary for each case. Careful dissection is performed by keeping the mucosal and submucosal layers of the vaginal wall. After a complete release of the mucosal flap, the ventral portion of the urethral obstruction, as well as the area of fibrosis immediately proximal and distal to the obstruction, was incised, preserving the urethral meatus (Figure 1).

**Figure 2:**
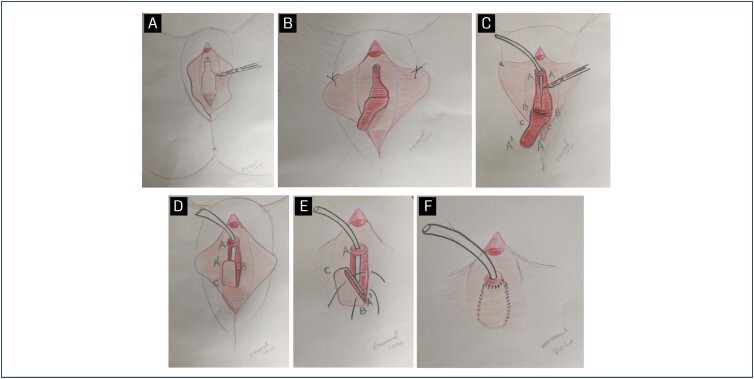
Step-by-step illustrations

Urethral reconstruction was performed with the anastomosis of the distal portion of the flap with the proximal portion of the urethral opening. A continuous suture was performed with a 4-0 absorbable monofilament (polyglecaprone 25), creating a neo-urethral path. After completing the urethral suture, the flap edges were sutured to the vaginal edges with a 3-0 absorbable multifilament continuous suture (Figure 2).

Patients were maintained with a 14Fr urethral foley catheter for 3 weeks, which was withdrawn in the female urology outpatient clinic. Follow-up was performed at 2, 4, 6, and 12 months postoperatively and then yearly, with all patients undergoing uroflowmetry with PVR evaluation 6 months after the procedure. During outpatient consultations, patients were questioned about the following lower urinary tract symptoms: weak stream, incomplete emptying sensation, urinary urgency, incontinence, and UTI.

### Statistical analysis

For all qualitative variables analyzed, the incidence was calculated and represented as a percentage. For the quantitative variables Qmax, PVR, and Pdet.max, the mean, minimum, and maximum values were calculated. Although the quantitative variables (Qmax and PVR) are normally distributed, a Wilcoxon test was used because of the number of patients analyzed. We defined an objective therapeutic success as a Qmax >12mL/s and the ability to place a urethral catheter bigger than 14 Fr.

## Results

Nineteen patients with a mean age of 56.4 years (range 34-80) and an average follow-up time of 21.4 months (8-70 months) were submitted to this procedure. The main causes of urethral stricture were endoscopic manipulation (n=6, 31.5%) and idiopathic stenosis (n=5, 26.3%) (Table 1). Two patients presented with urethral neoplasia in anatomical pathology exam. Most patients had undergone urethral dilatation as primary treatment before the urethroplasty (n=11, 57.8%).

**Table 1:** Demographic data of the patients

Total patients - n		19
Age in years - mean (range)		56.4 (34-80)
Previous dilatation - n (%)		11 (57.8%)
Causes - n (%)		
	Endoscopic procedure / Iatrogenic	6 (31.5%)
	Primary stenosis	5 (26.3%)
	Urethral neoplasia	2 (10.5%)
	Labor complications	2 (10.5%)
	Radiotherapy	1 (5.0%)
	Lichen	1 (5.0%)
	Urethral polyp	1 (5.0%)
	Trauma	1 (5.0%)
Mean follow-up - months (range)		21.4 (8-70)

The most commonly self-reported symptoms were voiding effort (n=19, 100%), weak stream (n=18, 94.7%) and recurrent UTI (n=7, 36%). One patient reported urgency associated with incontinence. In our series, no patient had renal failure. Among all patients, we are able to retrieve only 11 patients who underwent a preoperative voiding cystourethrogram, which showed an anatomical obstruction. In the urodynamic room, all patients were catheterized with a catheter no larger than 14Fr to avoid urethral dilatation. The mean (range) preoperative Pdet.max was 70.63cmH_2_O (40-110), and the preoperative Qmax was less than 12mL/s for the entire cohort. The mean surgical time was 75min (range 64 - 120min), with no patients having significant measurable bleeding (i.e. <100ml). In all those cases, there weren’t any other concurrent surgeries performed. Comparing the data from the preoperative (preop) and postoperative (postop) uroflowmetry and PVR, we find that the mean (range) Qmax increased significantly from 5.3mL/s (2-8) to 14.7mL/s (5-21) (p≤0.05), and the mean PVR volume decreased significantly from 101.4mL (34-480) to 47.3mL (0-200) (p≤0.05) (Table 2).

**Table 2:** Urodynamic and clinical results

	Preop	Postop	p-value
Urodynamic data			
Qmax (mL/s)	5.3 (2-12)	14.6 (5-21)	(p≤0.05)
PVR (mL)	101.4 (0-480)	47.4 (0-200)	(p≤0.05)
Clinical data (%)			
Straining	100	15.7	(p≤0.05)
Weak stream	94.7	10.5	(p≤0.05)
Recurrent UTI	36	5.2	(p≤0.05)
Incomplete emptying	100	15.7	(p≤0.05)

Of the 7 patients with recurrent UTI, 6 didn’t have any UTI 24 months after surgery. Voiding effort and weak stream were not reported in 16 and 17 of the 19 patients, respectively. One patient had urinary incontinence associated with urgency, which had been observed before the procedure. Three patients did not achieve therapeutic success, experiencing persistent low urinary flow (Qmax <12mL/s), narrow urethra, and high PVR. The etiology of these patients’ strictures were due to endoscopic procedures (two cases) and lichen. The success rate of the procedure was 84.2% (a total of 16 of 19 patients), and among them, the mean Qmax was 17.5mL/s, the PVR was 22mL and nobody complained of weak stream, voiding effort, or incomplete emptying.

## Discussion

FUS is an uncommon diagnosis, mostly because it is a lesser-known disease in clinical practice and presents with variable urinary tract symptoms.^(11)^ It is estimated that up to 8% of women with an infravesical obstruction have urethral stricture.^(12)^

The diagnostic criteria used (low Qmax and high detrusor voiding pressure) were based on the urodynamic diagnosis of infravesical obstruction in women.^(3)^ The measurement of the female urethral gauge varies according to the reference and usually supports a urethral catheter greater than 17Fr. For the characterization of urethral stricture, we utilized a urethral diameter less than 14Fr as a reference, as described by Smith et al.^(13)^

The main causes for stricture reported in the literature have been summarized by Osman et al.,^(2)^ in the order of frequency: idiopathic, iatrogenic, and traumatic. In our series, we observe a similar distribution of causes, but the prevalence of iatrogenic cases was followed by that of primary narrowing without any previous manipulation. Another recent review also found a profile of causes similar to those found in this study.^(14)^

Urethroplasty has been gaining ground as the standard treatment due to a greater knowledge of the female anatomy and more specialized services for treating voiding dysfunctions. These developments allow for additional clinical suspicion and increased FUS diagnoses. In the last three decades, numerous techniques have been described, all with good success rates, but none as a gold standard.

The dorsal approach provides good vascularization and anatomical support for the flaps or grafts and is the most commonly used technique. However, due to the omega shape of the urethral sphincter, there is a chance of injury, which could lead to incontinence.^(5)^ The ventral approach using a vaginal mucous flap uses an available and easily obtainable mucosa.^(15)^

Distinct from the original technique, we chose to preserve the urethral meatus, as we believe it results in a lesser chance of secondary hypospadias generating intra-vaginal urine flow.^(10)^ We did not find any study with the meatal sparing technique, but it is supposed that the role of the normal size meatus is important to keep a regular shape in the urine flow during micturition. In the male urethra, this has been a topic in discussion lately, and we believe this might also have an important role in the female urine flow. So, to keep it more anatomical and physiological, we preserve the meatus in its original form.

Although stress urinary incontinence is a concern in patients undergoing urethroplasty, it is uncommon. In our series, no patient presented this complication. If this complication occurred, the correction would be performed conventionally, with the placement of an autologous suburethral sling as performed in patients without previous surgeries, or a Burch colposuspension. In a review by Osman in 2012,^(2)^ de novo incontinence was not observed in patients undergoing ventral urethroplasty.

Our patients presented statistically significant uroflowmetry improvements in Qmax elevation and PVR reduction (p≤0.05). Although we did not use any standardized questionnaire to measure urinary tract symptoms, improvement of self-reported symptoms was significant, with most patients presenting with an improvement in symptoms. Our findings are comparable to those described in the largest series ever published.^(16,17)^

There was also an important improvement in the flow rate, and, even if most women did not reach peak flow higher than 25ml/s, we considered the improvement of symptoms and flow rate sufficient to determine the success of the method. We believe that the objective of the surgery is to improve symptoms and quality of life and, for that, we do not necessarily need a peak flow higher than 25ml/s.

The therapeutic failure we observed in 3 patients resulted in a success rate comparable with that described in the literature for similar techniques (83-100%). Our group had heterogeneous etiologies and strictures lengths, which might impact the results.^(18,19)^

We believe this to be a relevant paper on the subject of a rare condition such as female urethral strictures. Our sample size is adequate, considering the rarity of this condition, and similar to other previous publications. Herein, we present a feasible technique, which we believe can be easily replicated in other centers. However, this study is not without limitations. We do not have a control group to compare our technique because of the low sample size. Also, our surgeries are performed by residents, but the surgical staff was the same on all procedures; therefore, this must be studied in other centers to confirm our results.

## Conclusion

Although there is no consensus regarding the best approach for the correction of female urethral stricture, we believe that the ventral urethroplasty with meatal preservation technique described herein may be an alternative, with a good success rate and a significant improvement in uroflowmetry patterns. The advancement in female urology subspecialty in the last decades has made more techniques for the treatment of female urethral stricture to be described. Despite this, further studies are needed to define the proper management of the disease.
